# Addition of Pericapsular Nerve Group and Transversus Abdominis Plane Blocks Significantly Reduces Opioid Use in Patients Undergoing Concomitant Hip Arthroscopy and Periacetabular Osteotomy

**DOI:** 10.7759/cureus.33277

**Published:** 2023-01-02

**Authors:** Sean Ellis, Joshua D Harris, Derek P Flemming, Thomas J Ellis, Robert C Kollmorgen

**Affiliations:** 1 Orthopedics, Orthopedic One, Columbus, USA; 2 Orthopedics and Sports Medicine, Houston Methodist Hospital, Houston, USA; 3 Anesthesia, OhioHealth, Columbus, USA; 4 Orthopedic Surgery, Orthopedic One, Dublin, USA; 5 Hip Preservation and Sports Medicine, University of California, San Francisco, Fresno, USA

**Keywords:** postoperative pain, peripheral nerve block, hip arthroscopy, periacetabular osteotomy, transverse abdominis plane block, pericapsular nerve group block

## Abstract

Introduction: Previous studies have evaluated the effect of the pericapsular nerve group block for hip arthroscopy and the transverse abdominis plane block for periacetabular osteotomy and have shown decreased narcotic consumption in both groups. No published study has evaluated the effectiveness of combining the blocks when performing hip arthroscopy and periacetabular osteotomy under the same general anesthesia. It was hypothesized that patients treated for hip dysplasia with hip arthroscopy and concomitant periacetabular osteotomy using a pericapsular nerve group block, transverse abdominis plane block, and general anesthesia would have decreased postoperative pain and require less narcotic consumption than those undergoing the procedure with general anesthetic alone.

Methods: A single surgeon performed a retrospective analysis of consecutive patients undergoing concomitant hip arthroscopy and periacetabular osteotomy between 11/2020 and 6/2021. Fifteen consecutive patients undergoing the procedure with a general anesthetic alone (no-block group) were compared to 15 patients undergoing the same procedure with a combined pericapsular nerve group block, transverse abdominis plane block, and general anesthetic (block group). Hip arthroscopy was performed utilizing a post-free technique, and a rectus sparing approach was used for the periacetabular osteotomy. The nerve blocks were performed by multiple anesthesiologists using previously published methods. Operating room time, length of stay, visual analog scale pain scores, and total narcotic consumption in morphine milliequivalents were analyzed. Groups were compared using the chi-squared test for non-continuous demographic variables and a two-tailed t-test for continuous variables utilizing Microsoft Excel (Microsoft, Redmond, WA, USA), p-value set at 0.05 for significance.

Results: The no-block group consisted of 14 females and one male, while the block group was all females. No significant differences were observed between age, sex, BMI, surgery time, length of stay, or procedures performed, p>0.05. The maximal visual analog scale score in the post-anesthesia care unit was 8 ± 1.3 vs. 7 ± 1.9 in the no-block vs. block groups, respectively, p=0.15. The average hospital floor visual analog scale score was 5.7 ± 1.3 vs. 4.8 ± 1.3 in the no-block vs. block groups, respectively, p=0.07. Total pain medications required were 217.6 ± 54.6 vs. 154 ± 41.9 morphine milliequivalents in the no-block vs. block groups, respectively, p=0.001. No complications were reported in either group, and no patient in the block group demonstrated motor nerve palsy or postoperative fall.

Conclusion: This study demonstrated that patients undergoing combined hip arthroscopy and periacetabular osteotomy for symptomatic acetabular dysplasia who had pericapsular nerve group, transverse abdominal plane block, and general anesthesia required fewer narcotics in the first 24 hours after surgery compared to those who had general anesthesia alone.

## Introduction

Acetabular dysplasia (AD) is a common cause of hip pain in young adults [1,2]. In patients who are nonresponsive to non-surgical care, periacetabular osteotomy (PAO) is an accepted surgical treatment with good long-term outcomes in non-arthritic individuals [3,4]. More recently, hip arthroscopy has been combined with PAO (HA+PAO), with HA used to address labral tears and cam morphology [5]. In a small series, Maldonado et al. found that patients who underwent combined HA+PAO with a minimum five-year follow-up had significant improvement in patient-reported outcome scores. 

As understanding of AD and the role of PAO continues to evolve, perioperative strategies to improve pain and reduce opioid use are needed. Anesthesia management varies from general endotracheal anesthesia (GETA) alone to combined GETA with regional techniques, including epidural, spinal anesthesia, and peripheral nerve blocks (PNB) [6-8]. Regional anesthesia has been shown to decrease acute postoperative pain and opioids consumed in the perioperative period. The optimal modality to treat perioperative pain following HA+PAO has yet to be determined. 

Transversus abdominis plane (TAP) block and the pericapsular nerve group (PENG) block have been independently reported on as regional anesthetic options for hip preservation procedures [7,8]. The TAP block is designed to block the lower intercostal nerves as well as the ilioinguinal and iliohypogastric nerves and reduces perioperative pain following abdominal procedures. In a randomized trial of patients undergoing PAO, Löchel et al. demonstrated that the TAP block reduced postoperative pain compared to GETA alone [7]. The PENG block has also been utilized in patients with femoroacetabular impingement syndrome (FAIS) that underwent HA and has shown significant reductions in perioperative pain and opioid use [8-14]. The PENG block is performed under an ultrasound-guided technique allowing local anesthetic to be injected into the superior pubic ramus near the iliopectineal bursa [12,13]. The block targets the branches of the femoral and obturator nerves. 

While small series have demonstrated decreased opioid use and pain levels when using the PENG block for HA and the TAP block for PAO, there are no published studies evaluating the efficacy of combined PENG/TAP block for concomitant HA+PAO procedures. The purpose of this study is to retrospectively compare opioid use, pain, and length of hospital stay between a consecutive series of patients undergoing combined HA+PAO treated with a GETA alone to patients treated with combined GETA+PENG/TAP. We hypothesize that the addition of the PENG/TAP blocks to GETA will significantly reduce total opioid use, mean pain values using Visual Analog Scale (VAS) scores, and length of stay compared to GETA alone. 

## Materials and methods

Approval was obtained through Mt. Carmel Institutional Review Board (#220216-3). A retrospective analysis of prospectively collected data was executed for consecutive patients who underwent HA, labral repair, and femoroplasty with concomitant PAO from November 2020 to June 2021. HA + PAO was performed by a single high-volume hip preservation surgeon (TE), and PENG and TAP blocks were performed by multiple anesthesiologists with experience in regional anesthesia. Primary outcome measures were the VAS score (cm) and opioid use (measured via morphine milligram equivalents [MME]) during the first 24 hours after admission to the floor post-procedure. The secondary outcome was any block-related complications.

Inclusion criteria were patients undergoing primary hip arthroscopy, labral repair, and femoroplasty with concomitant PAO for hip dysplasia with Tönnis 0 or 1-grade radiographs. The indication for labral repair was an unstable labrum identified at the time of the hip arthroscopy. Femoroplasty was performed for an alpha angle greater than 50 degrees. The indication for PAO was determined by assessing for under coverage of the femoral head as measured by acetabular and femoral radiographic parameters and the presence of joint hypermobility. Patients were excluded if they did not have both a labral repair and femoroplasty or were undergoing revision HA or revision PAO. 

PENG/TAP blocks were performed using a standard ultrasound-guided technique in the preoperative holding area. The GETA group underwent a general anesthetic only, and no local anesthetic was otherwise given in either group. PENG/TAP blocks were performed with the patient in the supine position. For the PENG block, the ultrasound probe was placed over the anterior inferior iliac spine (AIIS) and rotated 45 degrees to be in position with the pubic ramus. The needle was inserted using an in-plane method to ensure needle placement was between the pubic ramus and the psoas tendon. Using a 22-gauge (g) needle, 20 milliliters (mL) of ropivacaine was injected. The ultrasound probe was used and placed laterally over the TAP plane for the TAP block. After visualization of the TAP plane, the needle was inserted using an in-plane method to ensure needle placement was between the internal oblique and transversus abdominis muscle. Using a 22-g needle, 20 mL of ropivacaine was injected. The no-block (NB) group received only GETA.

The hip arthroscopy portion of the procedure was performed before the PAO. It utilized the modified supine position with a Smith and Nephew (Andover, MA, USA) distraction table with a previously described post-free technique [15]. Standard anterolateral, mid-anterior, and distal anterolateral portals were utilized. Labral repair and femoroplasty were performed as indicated, and the capsule was meticulously closed in all patients. Next, patients were transferred to an OSI (Union City, CA, USA) flattop radiolucent table. The PAO was performed via a standard rectus-sparing approach [16]. Once the fragment was mobilized and repositioned, it was secured in all cases with four 4.5 mm fully threaded cortical screws placed from the iliac wing into the periacetabular fragment. Following the PAO, to address subspine impingement, if present, the rectus femoris direct head tendon was detached from the AIIS from medially to laterally, and a 5.5 mm burr was used to resect the prominent portion of the AIIS, and the tendon was then repaired through drill holes to the remnant of the AIIS. 

Post surgery patients were admitted to a standard medical surgical unit. Physical therapy was initiated postoperative day zero for 20-pound foot flat gait training, and pain management was performed with IV and oral pain medication. This partial weight bearing was maintained for six weeks post surgery. 

Outcome measures from the start of the procedure through 24 hours after discharge from the post-anesthesia care unit (PACU) were extracted from chart review: VAS maximum in the PACU (cm), mean floor VAS (cm) score, total MME, and length of stay (days). VAS has been validated to measure pain levels and has been previously validated for hip arthroscopy to measure chronic and acute pain [17]. The nursing staff documented VAS in the PACU on a Likert scale, 0-10, based on the question, “How much pain do you have in your hip?” A measurement of 0 was no pain, while a measurement of 10 was the worst pain possible. Two measurements of VAS were extracted from the chart: VAS Maximum (maximum documented PACU score) and the average of VAS collected from admission to the floor postoperatively through 24 hours post-admission. To standardize reported opioid usage, all opioids administered were converted to MME.

Microsoft Excel (Microsoft, Redmond, WA, USA) was used to perform statistical analysis. Groups were compared using a chi-squared test for non-continuous demographic variables and a two-tailed t-test for continuous variables utilizing Microsoft Excel, p-value set at 0.05 for significance. An a priori power analysis was performed to detect a 2-point difference in the VAS and affirmed that 17 patients would be required in each group to observe a p<0.05 significance with 1-beta = 0.8. Fifteen consecutive patients undergoing PENG/TAP were matched to 15 consecutive NB patients retrospectively. Demographics of age, sex, and body mass index (BMI) were obtained from a chart review. 

## Results

Thirty consecutive patients met the inclusion criteria. The no-block group consisted of 14 females and one male, while the block group had 15 females. Demographics are shown in Table 1. No significant differences were observed between age, sex, BMI, surgery time, length of stay, or procedures performed (Table 1). All patients underwent identical procedures (Table 2). VAS Max in the PACU was 8 ± 1.3 vs. 7 ± 1.9 in the no-block vs. block groups, respectively, p=0.15. VAS Floor was 5.7 ± 1.3 vs. 4.8 ± 1.3 in the no-block vs. block groups, respectively, p=0.07. Opioid consumption was 217.6 ± 54.6 MME vs. 154 ± 41.9 MME in the no-block vs. block groups, respectively, p=0.001 (Table 3). No complications were reported in either group, and no block group patient demonstrated motor nerve palsy or postoperative fall.

**Table 1:** Demographics of cohort NB (no block), PENG (pericapsular nerve group), SD (standard deviation), TAP (transverse abdominus plane), BMI (body mass index), CI (confidence interval), p-value significant < 0.05.

		PENG/TAP		NB			
	mean	SD	95% (CI)	mean	SD	95% (CI)	p-value
Age (years)	21.92	5.35	18.97 - 24.88	23.92	6.9	20.09 - 27.74	0.38
Sex (% Female)	100	15/15		93.33	14/15		
BMI (kg/m^2^)	23.9	2.9	22.3 – 25.5	24.9	3.8	22.8 – 27.1	0.4

**Table 2:** Procedures performed NB (no block), PENG (pericapsular nerve group), TAP (transverse abdominus plane), PAO (periacetabular osteotomy), p-value significant < 0.05.

Procedures	PENG/TAP	NB	p-value
Arthroscopic Femoroplasty	15	15	1
Arthroscopic Labral repair	15	15	1
Arthroscopic Capsular Repair	15	15	1
Open Anterior Inferior Subspine Decompression	15	15	1
PAO	15	15	1

**Table 3:** Outcome measures NB (no block), PENG (pericapsular nerve group), TAP (transverse abdominus plane), BMI (body mass index), CI (confidence interval), SD (standard deviation), p-value significant < 0.05 in bold. OR (operating room), VAS (visual analog scale), LOS (length of stay), MME (morphine milligram equivalence).

		PENG/TAP		NB			
	mean	SD	95% (CI)	mean	SD	95% (CI)	p-value
OR Time (minutes)	267.2	21.48	255.3 - 279.09	272.33	30.41	255.49 - 289.17	0.58
VAS Max PACU	7.06	1.98	5.96 - 8.16	8	1.35	7.21 - 8.78	0.15
VAS Floor	4.85	1.35	4.1 - 5.6	5.78	1.36	5.02 - 6.54	0.07
LOS days	1.4	0.69	1.02-1.79	1.56	0.92	1.04 - 2.07	0.61
Total MME	154.03	41.99	130.77 - 177.28	217.6	54.67	187.32 - 247.87	0.001

## Discussion

The most important finding of this study demonstrated that patients with combined GETA+PENG/TAP used significantly less opioids than those with GETA alone. There were no block-related complications. There are no previously published studies reporting on the efficacy of a combined PENG/TAP block for a HA/PAO procedure. This study suggests that the additional blocks are safe and can aid perioperative pain management. 

The TAP block for PAO was initially described by Löchel et al. [7]. They reported on 42 consecutive patients undergoing PAO surgery who were randomized to receive either GETA alone or GETA/TAP. Total opioid consumption was significantly less in the TAP group at six, 24, and 48 hours postoperatively. The average pain score was significantly less at 24 hours postoperatively. The PENG block for hip arthroscopy using a post-free technique was previously described by Kollmorgen et al. [15]. They reported on 25 patients with a GETA/PENG and 25 patients with GETA alone. The GETA/PENG group had significantly less intraoperative fentanyl use, total MME, shorter time to discharge, and lower initial PACU VAS. 

Many surgeons use epidural anesthesia following PAO to assist with postoperative pain control, though there is limited published data on its efficacy. Cunningham et al. compared patients who had the epidural removed postoperative day one versus day two [6]. The early removal group had significantly lower pain scores and narcotic usage. Fascia iliacus block has also been described following PAO. Albertz et al. compared 16 patients with a fascia iliacus block to 16 patients with an epidural following PAO for AD [18]. The fascia iliacus block group had significantly shorter LOS and lower MME. Pain control and early mobilization are the two primary factors limiting early discharge following PAO. Both epidural and fascia iliacus regional anesthesia can cause significant short-term muscle weakness, which delays mobilization. The combined TAP/PENG block significantly reduced narcotic requirements without blocking motor function, allowing early mobilization. Additional studies are needed to determine if this approach may decrease LOS. 

This study is not without limitations. Given the small number of subjects, the primary outcome measure VAS pain comparison may be underpowered. The data collected other than LOS is limited to 24 hours after discharge from the PACU rather than for the entire hospitalization. This time point was chosen since a significant number of patients were discharged within 24 hours. Finally, it is a single surgeon study which may limit general applicability. One strength of the study is that the surgeries included in the study were performed over a five-month window, and the perioperative protocols were consistent during the study. 

## Conclusions

Decreased opioid consumption is paramount for patient well-being. This study demonstrated that patients undergoing HA/PAO for symptomatic AD and FAIS who had combined GETA/PENG/TAP anesthesia utilized significantly less opioids in the first 24 hours after surgery compared to a matched control group who had GETA alone. No significant differences were observed in any other outcome measure studied. Future prospective randomized studies addressing the effect of PENG/TAP with PAO surgery on opioid use and pain beyond the first 24 hours after surgery are needed to better understand the utility of these regional anesthetic modalities.
